# Hypertrophic cardiomyopathy mutations in the calponin-homology domain of ACTN2 affect actin binding and cardiomyocyte Z-disc incorporation

**DOI:** 10.1042/BCJ20160421

**Published:** 2016-08-11

**Authors:** Natalie J. Haywood, Marcin Wolny, Brendan Rogers, Chi H. Trinh, Yu Shuping, Thomas A. Edwards, Michelle Peckham

**Affiliations:** * Astbury Centre for Structural Molecular Biology and the School of Molecular and Cellular Biology, Faculty of Biological Sciences, University of Leeds, Leeds LS2 9JT, U.K.

**Keywords:** actin, α-actinin, cardiomyocytes, crystal structure, familial hypertrophic cardiomyopathy, imaging

## Abstract

α-Actinin-2 (ACTN2) is the only muscle isoform of α-actinin expressed in cardiac muscle. Mutations in this protein have been implicated in mild to moderate forms of hypertrophic cardiomyopathy (HCM). We have investigated the effects of two mutations identified from HCM patients, A119T and G111V, on the secondary and tertiary structure of a purified actin binding domain (ABD) of ACTN2 by circular dichroism and X-ray crystallography, and show small but distinct changes for both mutations. We also find that both mutants have reduced F-actin binding affinity, although the differences are not significant. The full length mEos2 tagged protein expressed in adult cardiomyocytes shows that both mutations additionally affect Z-disc localization and dynamic behaviour. Overall, these two mutations have small effects on structure, function and behaviour, which may contribute to a mild phenotype for this disease.

## Abstract (precis)

We have discovered that two mutations at the actin binding domain (ABD) of α-actinin-2 (ACTN2), which cause hypertrophic cardiomyopathy (HCM), have minor effects on its structure and ability to bind actin and integrate into Z-discs, providing a potential disease mechanism.

## INTRODUCTION

Familial hypertrophic cardiomyopathy (HCM) is a cardiovascular disorder affecting between 1 in 200 and 1 in 500 people in the general population [1,2]. It is commonly characterized by a hypertrophic left ventricle, and an impaired diastolic relaxation of the heart [3]. Currently well over 1000 mutations in a range of sarcomeric proteins have been identified that cause HCM, with over 500 mutations in the gene for β-cardiac myosin heavy chain (*MYH7*) alone [4]. Mutations in MYH7 and cardiac myosin-binding protein C cause approximately 70% of the familial HCM cases [5]. Mutations in several proteins found in the Z-disc have been implicated in HCM [6], including the recent discovery of mutations in α-actinin 2 (*ACTN2*) [7]. *ACTN2* is one of four genes encoding isoforms of α-actinin, two of which (*ACTN1* and *ACTN4*) encode non-muscle isoforms. The third gene (*ACTN3*) is co-expressed with *ACTN2* in skeletal muscle, whereas only *ACTN2* is expressed in cardiac muscle [8].

**Figure 1:**
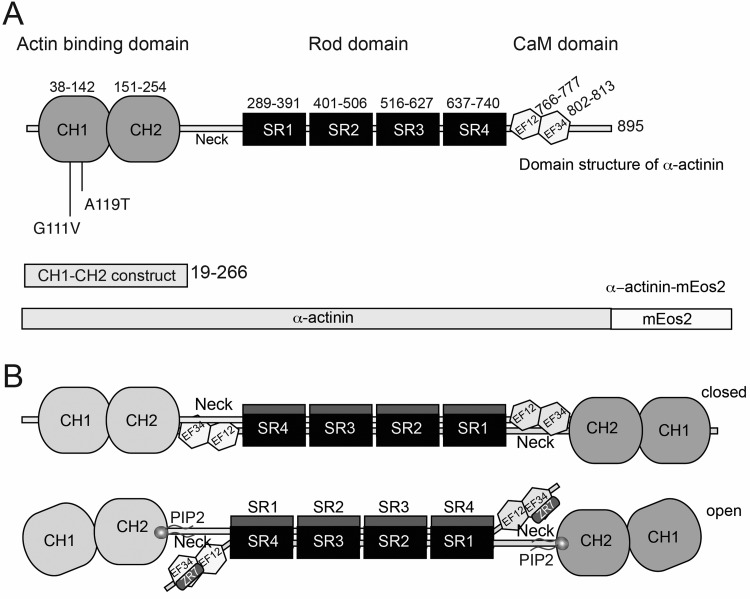
Domain structure of ACTN2 (**A**) The domain structure of α-actinin, showing the positions of the different domains. The positions of the two HCM mutations in the ABD studied here are indicated. The ABD is made up of 2 CH domains. The calmodulin-like domain (CaM) contains two pairs of EF hands (EF12 and EF34). Numbers refer to amino acid residues for human skeletal ACTN2. The two main expression constructs used in the present study are shown below. (**B**) The molecule forms an antiparallel homodimer with a ‘closed’ conformation in the absence of PIP_2_, in which the CaM domains interact with the ABD domain. On the binding of PIP_2_, the molecule adopts an open conformation which allows ACTN2 to bind to actin via its ABD, and to the ZR7 of titin via its second CH domain (EF34) in the Z-disc.

All four isoforms of α-actinin consist of an N-terminal actin binding domain (ABD) comprising two calponin homology (CH) domains, connected via a flexible linker, which allows for conformational flexibility in the ABDs, to a rod domain (Figure 1A). The rod domain contains four spectrin repeats (triple helix coiled-coil bundles; SR1–4). The C-terminus of the protein contains a calmodulin-like domain (CaM) composed of four EF hands, a well characterized Ca^2+^ binding motif [9]. The rod domain is 24 nm long, and both acts as a stiff spacer and is responsible for the formation of an antiparallel homodimer. However, the EF hands in striated muscle specific isoforms of ACTN have lost their ability to bind Ca^2+^ and binding to actin is instead regulated by PtdIns(4,5)*P*_2_ (PIP_2_) to the ABD [10–12]. Binding of PIP_2_ has been shown to activate ACTN2 by promoting the ‘open’ conformation of the molecule (Figure 1B), and increasing its ability to bind to Z-disc titin motifs [13]. Thus *ACTN2* is important in cross-linking actin and titin filaments in the Z-disc [14]. A crystal structure for human ACTN2 was recently reported, confirming that this molecule forms an antiparallel heterodimer (as diagrammed in Figure 1B) with actin binding sites (ABDs) at either end, enabling the protein to bundle and cross-link actin filaments [13].

Eight distinct mutations in ACTN2 have been reported [7], yet despite its pivotal role in the Z-disc, very little has been done to understand how these mutations lead to disease, in contrast with mutations in non-muscle isoforms such as ACTN4 [15,16]. For example, the kidney disease causing mutation, ACTN4-K225E, increased the binding affinity of ACTN4 for actin, leading to increased actin filament aggregation, providing an explanation for the disease [16].

Two mutations for the actin binding domain (ABD) on ACTN2 have been described: G111V ([17], which was associated with an HCM patient with a type of hypertrophy classified as ‘sigmoidal’ thought to be more typical of Z-disc linked disease; and A119T [7], as one of three mutations picked up in a screen of Australians with HCM. The original family reported to have an association of the A119T mutation with HCM, had a variable phenotype, with some patients only developing heart failure at the age of 75, and others showing heart failure at ages 36–44 [7]. G111V was only reported for a 31 year old male [17], with no family history of HCM. Although he did have significant myopathy it is possible that there were other undetected mutations that may have contributed to his disease. It is not known how these mutations affect the properties of ACTN2.

To determine how these two mutations might contribute to HCM, we have expressed and purified the ABD comprising the two CH domains from human ACTN2, together with two mutant variants A119T (AT) [7] and G111V (GV) [7,17] and determined their structure, and actin binding affinity. In addition, we investigated the effects of the mutations on the localization and dynamic behaviour of full length ACTN2 tagged with mEos2 in cardiomyocytes.

## EXPERIMENTAL

### Cloning of α-actinin-2 constructs

The DNA coding sequence for human α-actinin-2 (ACTN2) was purchased from OriGene as a GFP fusion protein in a pCMV6-AC-GFP plasmid (RG208531). The full length open reading frame was subcloned into a modified pEGFP-N1 plasmid, in which the mEos2 [18] replaced eGFP, the *NotI* site following mEos2 was replaced with a *SalI* site, the *XhoI* site in the multiple cloning site (MCS) had been deleted and a novel NotI site introduced upstream of mEos2 to enable cloning of the cDNA for ACTN2 between the *BamH1* and *NotI* site in frame with mEos2 ACTN2. ACTN2-mEos2 was then subcloned into the Adeasy pdc315 plasmid using *EcoRI* and *SalI*. After confirmation that the sequence was correct [Sanger sequencing (GATC Biotech)], this plasmid was then used to generate adenovirus for expression in adult rat cardiomyocytes as described [19]. mEos2 was a kind gift from Michael Davidson and Loren Looger [18]

To generate the pGEX6p-1 plasmid with ACTN2 CH domains, DNA for amino acids 19–266 was amplified by PCR. *EcoRI* and *SalI*/stop sites were introduced using the primers (5′ and 3′ respectively). The PCR products were subcloned into a TOPO vector, and sequenced. The TOPO vectors were then digested with *EcoRI* and *SalI* and the cDNA for the CH domains ligated into pGEX-6p-1, in frame with glutathione transferase (GST).

### Generation of mutants

Overlap PCR was used to generate point mutations. Short fragments containing the desired mutations were subcloned into the pdc315 ACTN2-mEos2 plasmid to replace the wild type (WT) sequence and generate mutant full length constructs for expression using the adenovirus system. Overlap PCR was also used to generate mutant constructs for expression using the pGEX6P1 plasmid. All plasmids were Sanger sequenced (GATC Biotech) to check that the ACTN coding sequences were correct.

### Expression and purification of CH domains

WT α-actinin binding domain (ABD) and its mutants (A119T; G111V) were expressed in *Escherichia coli* BL21 Rosetta 2 (Novagen). Overnight small volume (5 ml) cultures were used to inoculate 400 ml LB medium. Cultures were grown for 3 h in 37°C, 220 rpm shaking until reaching *D*_600_ ∼ 0.6 when they were induced with 0.5 mM IPTG and grown for additional 3 h in the same conditions. Harvested pellets were resuspended in the lysis buffer (PBS, 1 mM DTT, 1 mM EDTA, 200 μg/ml lysozyme, 0.1% Triton X-100, 1 protease inhibitors tablet/50 ml) and stored at −80°C.

Proteins were purified using GST-tag affinity chromatography on SuperGlu resin (Generon). Thawed lysates were sonicated (6 cycles of 10 s on/off pulses), centrifuged (30000***g***, 20 min) and applied on to the equilibrated column. Column was washed five times with PBS and bound protein was incubated with prescission protease in cleavage buffer (50 mM Tris, 150 mM NaCl, 1 mM EDTA, 1 mM DTT, pH 7.5) overnight in 4°C on a roller. After cleavage 8×1 ml fractions were collected and the column was regenerated using 15 mM reduced glutathione. The purity of the fractions was assessed by SDS/PAGE. Pooled fractions were either dialysed against CD buffer (100 mM NaCl, 10 mM sodium phosphate, pH 7.4) for CD measurements, or against F-actin buffer (50 mM KCl, 10 mM MOPS, 1 mM EGTA, 1 mM MgCl_2_, 1 mM DTT, pH 7.0), for actin binding assays. Alternatively purified protein in the cleavage buffer was used for protein crystallization at a concentration of 10 mg/ml.

### Actin binding assays

Skeletal muscle actin was polymerized from stocks of G-actin, purified from rabbit muscle as described [20]. Actin (∼70 μM) in buffer A (2.0 mM Tris pH 8.0, 0.2 mM ATP, 0.2 mM CaCl_2_, 1 mM DTT, 0.5 mM NaN_3_) was mixed on ice with exchange buffer (3 mM MgCl_2_, 11 mM EGTA) in a 10:1 ratio and after 5 min an equal volume of polymerization buffer (300 mM KCl, 12 mM MgCl_2_, 1 mM EGTA, 120 mM MOPS, pH 7.0) was added. After 2 h incubation actin was dialysed into F-actin (polymerization) buffer for 72 h in 4°C.

Polymerized actin (5 μM) was incubated for 1 h at room temperature with increasing concentrations of ABD and its mutants (2.5–60 μM) and spun-down, 100000 ***g***, 4°C, 30 min. Supernatant was collected and protein was precipitated using TCA whereas the pellet was resuspended in Laemmli buffer. Samples were applied on 15% SDS/PAGE gel and stained with Coomassie Blue. Image-Lab Bio-Rad software package was used to perform densitometry analysis. Control gels with actin or ABD in 1–60 μM concentration range were used for normalization of obtained data.

### CD spectroscopy

CD measurements were performed on an APP Chirascan CD spectropolarimeter with a 0.1 cm path length quartz cuvette in CD buffer (100 mM NaCl, 10 mM sodium phosphate, pH 7.4). Data were collected every 1 nm with 30 s averaging time, each measurement being an average of two repeated scans. Data presented are averaged data collected from at least two separate protein preparations. Thermal measurements were performed in a temperature range from 10 to 85°C with 0.7°C/min heating rate, data acquisition every 5°C and 20 s averaging time. The mean residue molar ellipticity of proteins was calculated as described previously [21]. Protein concentration was measured by absorption at 280 nm. Absorption coefficients were obtained from ProtParam software.

### Crystallization

Crystals were obtained using the hanging drop, vapour diffusion method with crystallization conditions consisting of different percentages and molecular mass PEG. Solution conditions were WT 30% PEG1500 (v/v), G111V mutant 28% PEG2000 (v/v) and A119T mutant 24% PEG3350 (v/v). The WT and mutant crystals grew at 18°C and belonged to the space group *P*1.

### Data collection and processing

Crystals were transferred to the crystallization buffer with the addition of 25% PEG400 (v/v) prior to flash freezing at 100 K. All ACTN2 data were recorded to a resolution range of between 1.9 and 2.0 Å (1 Å=0.1 nm) from single crystals at 100 K on the macromolecular crystallography beamline stations I02 and I04 at the Diamond Light Source. The diffraction images were integrated, scaled and reduced using two different sets of programs implemented as part of the CCP4 program suite [22] or Xia2 [23]. Among the data collected two different unit cell parameters were obtained *a*=38.7 Å, *b*=47.5 Å, *c*=70.3 Å, *α*=80.8°, *β*=80.8°, *γ*=76.5° for WT1 (AaB6II) and *a*=38.3 Å, *b*=46.6 Å, *c*=70.2 Å, *α*=73.8°, *β*=80.2°, *γ*=75.4° for WT2 (AaD2I), G111V mutant and A119T mutants; the differences being a change in the *α* angle of 7°. Five percent of the reflections from each of the WT data were selected at random and excluded from the refinement using the program FREERFLAG [24] and constituted the *R*_free_ set. The *R*_free_ set for G111V and A119T were copied from the dataset for WT2. The data processing statistics are shown in Table 1.

**Table 1:** Data collection, processing and refinement statistics for wild type ACTN2 and the mutants G111V and A119T

	WT1	WT2	G111V mutant	A119T mutant
Source	Diamond i04	Diamond i04	Diamond i04	Diamond i02
Wavelength (Å)	0.9464	0.9464	0.9796	1.0000
Resolution range (Å) *	68.80–2.00 (2.05–2.00)	67.00–1.90 (2.00–1.90)	67.14–1.88 (1.98–1.88)	66.68–2.01 (2.06–2.01)
Space group	*P*1	*P*1	*P*1	*P*1
Unit-cell parameters (Å)	*a*=38.7, *b*=47.5, *c*=70.3	*a*=38.3, *b*=46.6, *c*=70.2	*a*=38.2, *b*=46.5, *c*=70.4	*a*=38.4, *b*=46.6, *c*=69.9
	*α*=80.8, *β*=80.8, *γ*=76.5	*α*=73.8, *β*=80.2, *γ*=75.4	*α*=73.7, *β*=80.1, *γ*=75.5	*α*=73.8, *β*=80.0, *γ*=75.1
No. of observed reflections	67208	63654	71584	58524
No. of unique reflections	31249	33050	34585	28251
Redundancy	2.2 (2.0)	1.9 (2.0)	2.1 (2.1)	2.1 (2.1)
Completeness (%)*	96.4 (82.2)	93.6 (94.0)	94.2 (91.1)	95.0 (95.1)
<*I*/*σ*(*I*)>*	5.8 (1.5)	7.2 (2.1)	7.6 (2.0)	6.2 (2.2)
*R*_merge_ (%)* ^†^	12.2 (58.3)	9.0 (51.7)	7.6 (41.2)	7.7 (31.7)
*R*_pim_ (%)* ^‡^	12.1 (48.1)	7.4 (38.7)	7.1 (39.1)	6.5 (27.4)
Resolution range for refinement (Å)	68.80–2.00	67.00–1.90	67.14–1.88	66.68–2.01
*R* factor (%)	21.7	19.2	17.8	19.8
*R*_free_ (%)^§^	26.7	23.1	21.6	23.4
No. of protein non-H atoms	3723	3597	3547	3588
No. of water molecules	117	201	307	201
RMSD bond lengths (Å)^║^	0.012	0.009	0.007	0.011
RMSD bond angles (^◦^)^║^	1.4	1.3	1.1	1.4
Average overall *B* factor (Å^2^)				
Protein	30.5	26.4	20.7	23.3
Water	28.5	27.2	25.5	24.9
Ramachandran analysis, the percentage of residues in the regions of plot (%)^¶^				
Favoured region	98.0	98.7	98.2	98.4
Outliers	0	0	0	0
PDB code	5A36	5A38	5A37	5A4B

*Values given in parentheses correspond to those in the outermost shell of the resolution range.

†*R*_merge_=∑_*hkl*_∑_*i*_|*I_i_*(*hkl*) − ⟨*I*(*hkl*)⟩|/∑_*hkl*_∑*I_i_*(*hkl*).

‡*R*_pim_=∑_*hkl*_{1/[*N*(*hkl*) − 1]}^1/2^∑_*i*_|*I_i_*(*hkl*) − ⟨*I*(*hkl*)⟩|/∑_*hkl*_∑_*i*_*I_i_*(*hkl*).

§*R*_free_ was calculated with 5% of the reflections set aside randomly.

║Based on the ideal geometry values of Engh and Huber [37].

¶Ramachandran analysis using the programme MolProbity [38].

### Structure determination

Two crystal structures were obtained for WT ABD (WT1 and WT2, which refer to crystals from particular wells; AaB6II and AaD2I respectively), and one each for the two mutants. The crystal structure of WT2 was determined by molecular replacement (MR) using the program PHASER [25] with the human α-actinin-3 structure (PDB code 1WKU, [26] as the search model (94% sequence identity). One single MR solution was obtained from PHASER and after initial rounds of rigid body and restrained refinements using REFMAC5 [27], iterative cycles of manual model building using both 2*F*_o_-*F*_c_ and *F*_o_-*F*_c_ maps and refinement were carried out using COOT [28] and REFMAC5 respectively. Water molecules were manually added in COOT for peaks over 3.5*σ* in the *F*_o_-*F*_c_ map, and where appropriate hydrogen bonds could be made to surrounding residues or other water molecules. Refinement was judged complete when the *R* factor had converged and no significant interpretable features remained in the *F*_o_-*F*_c_ map. Similarly the WT1 structure was also determined by MR using PHASER with a single monomer of the refined structure of WT AaD2I as the search model. One single MR solution was obtained from PHASER and iterative cycles of manual model building and refinement carried out using COOT and REFMAC5. The refined WT2 structure was used as a starting model for the refinement of both the mutants in REFMAC5. The side chains of the mutated residues G111V and A119T were built into their respective *F*_o_-*F*_c_ maps and iterative cycles of manual model building and refinement carried out using COOT and REFMAC5. All structural validations were carried out with MOLPROBITY [29]. All refinement statistics are shown in Table 1. Coordinates and structure factor amplitudes for the structures have been deposited in the Protein Data Bank (www.pdb.org), and have been assigned PDB 5A36, 5A38, 5A37 and 5A4B.

### Cardiomyocyte culture and photoactivation experiments

Adult rat cardiomyocytes were isolated, and cultured as described [19] in glass bottomed dishes (Iwaki) coated with laminin. The cardiomyocytes were prepared from isolated rat hearts using a Langendorff retrograde perfusion technique with a collagenase-containing solution [30] by the Cardiovascular group at Leeds, using 20 week old male Wistar rats and kindly provided to us. Two separate experiments were performed for WT, G11V and A119T, and data from cells from both experiments used in the analysis. Virus was added to the cells in glass bottomed culture dishes (Ibidi) at an MOI (multiplicity of infection) of ∼50 as described [19] and the cells were incubated overnight before imaging.

The following day, the culture dishes were placed on a heated stage (37°C) of a confocal (Zeiss LSM510), and imaged with the Plan-achromat ×63, n.a.1.4, oil objective, using LSM Meta software and the multi-time series (MTS) macro. First, 5–7 cells in the dish were selected and their positions recorded. Next the zoom set to ×6, and for each cell, the image rotated so that the Z-discs in the cell were either vertical or horizontal in the frame. Once this was done, a separate configuration file was saved for each cell, and this was then loaded into the MTS macro. Next, for each cell, a small box or region of interest (ROI) [∼140×140 (6.6 μm) pixels; see Figure 6 for approximate position of the ROI prior to photoactivation] was positioned over the centre of the cell to select a region that would be illuminated with 405 nm light during the experiment. ROIs were individually positioned for each cell, and the positions saved in the MTS macro. The ROIs were slightly wider than three Z-discs. The ‘bleaching’ protocol was then set up such that the cells were imaged once before illuminating the box with 405 nm light (10 cycles, 22% laser power). The cells were then imaged every 150 s for at least 12 further frames, using 488 nm light (1–5% laser power) and 543 nm light (50% laser power). Laser intensities were chosen to minimize any bleaching, while providing suffient signal to noise, without the need for averaging. This experiment is similar to a photobleaching experiment, except that in this case the 405 nm light permanently switches ‘green’ fluorescent mEos2 molecules to ‘red’ within the ROI, Figure 6). The dynamic behaviour of the switched molecules was then measured from the decrease in red fluorescence in the initial ‘photoconverted’ region with time. This decrease reports on movement of ACTN2-Eos away from the region of photoconversion.

Additionally, cells were cultured on laminin coated glass coverslips as described [19], fixed using fresh warm 4% paraformaldehyde, which was added to culture medium (50:50) for 15 min. Cells were permeabilized using Triton-X 100 (0.5% in PBS) and co-stained for myosin using the A4.1025 antibody [19]. Fixed cells were imaged using a Deltavision deconvolution microscope equipped with a Roper CoolSnap Hq CCD camera, and using the 100× 1.4 N.A. Plan Apo lens. Identical image capture settings were used for all images.

Parallel cell cultures were used to make protein samples for SDS gels, which were then transfered to polyvinylidene fluoride membrane for use in Western blots, using antibodies to actin (as a loading control: Santa Cruz sc4778), ACTN (A5044; Sigma) and mEos (Badrilla). Secondary anti-HRP (horseradish peroxidase) antibodies, and Immobilon Western Chemiluminescent HRP substrate (Millipore) were used to detect antibody binding.

## RESULTS AND DISCUSSION

### Effects of mutations in the ABD on its secondary structure and thermal stability, and ability to bind to F-actin *in vitro*

**Figure 2:**
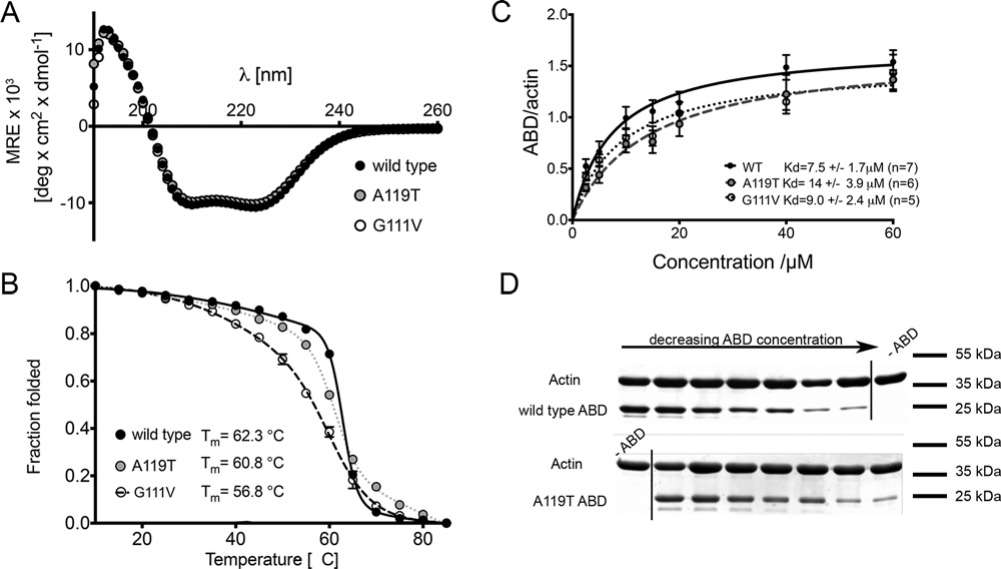
Analysis of the secondary structure, thermal stability and actin binding ability of the WT and mutant ACTN2 actin binding domain (ABD: CH1–CH2) construct (**A**) Far UV spectra show similar spectra for WT and the two mutant ABDs. Data shown are the mean values for three to four spectra (±S.E.M.) for each construct. (**B**) Thermal denaturation profiles of the WT and two mutant ABDs, together with the values at which half the protein has melted (*T*_m_). Data shown are the mean values (±S.E.M.) for three (WT, A119T) or two (G111V) experiments. (**C**) The binding curves for the WT and mutant ABD and actin. Data shown are the mean values (±S.E.M.) for at least five separate experiments. The mean *K*_d_ values (±S.E.M.) are shown in the legend. (**D**) Example gels for the pelleted F-actin together with co-pelleted WT ABD, and one of the mutant ABD (A119T) constructs. Arrow shows decreasing concentration of ABD. The gels include a lane without ABD as indicated.

Purified WT and mutant ABDs (AA19-266) from ACTN2 had a similar secondary structure as shown by circular dichroism (Figure 2A). All three constructs have almost identical far UV spectra with a strong α-helical signal (Figure 2A). The total helical content (measured by the molar ellipticity value at 222 nm) is similar for WT and both mutant constructs, suggesting that the mutations do not have a strong effect on secondary structure.

Thermal denaturation experiments showed that the thermal stability for both mutants was lower than that for the WT construct (Figure 2B). The G111V mutation had the greatest effect on stability with its melting temperature (*T*_m_=56.8°C) being 5.5°C lower than that for WT (62.3°C). The A119T mutation has an intermediate effect with *T*_m_ (60.8°C) only slightly less than WT (Figure 2B). These results indicate that although the mutations do not affect the secondary structure of the ABD, they lower its stability, with G111V mutation having the stronger effect.

Both G111V and A119T mutations reduced the binding affinity of the ABDs to F-actin. The *K*_d_ was increased ∼2-fold for the A119T mutant, and ∼1.2-fold for the G111V mutant constructs compared with the WT ABD (Figure 2C). However although we always observed a similar trend for these values, they were not significantly different. G111V lies just outside one of the three stretches of amino acid residues thought to mediate actin binding (residues 41–50, 116–140 and 145–165, reviewed in [31]) and A119T lies within one of these stretches. The measured *K*_d_ for the WT construct of 7.5 μM, is comparable to that measured for other ACTN ABDs [∼1 μM for smooth muscle ACTN (ACTN1), and 5 μM for *Acanthamoeba* ACTN [32]].

### Effects of mutations in the ABD on its structure

Given the small effects on secondary structure, we went on to crystallize the WT and mutant ABDs to determine what effect, if any, the mutations had on tertiary structure. We were successful in obtaining crystals for the WT and both mutant ABDs and were able to solve their structures to a resolution of between 1.9 and 2.2 Å (Table 1). The ABD was in the ‘closed’ conformation in all the structures, as found for other structures of the ABDs for the second striated muscle isoform ACTN3 [26], and the two non-muscle isoforms ACTN1 [33].

**Figure 3:**
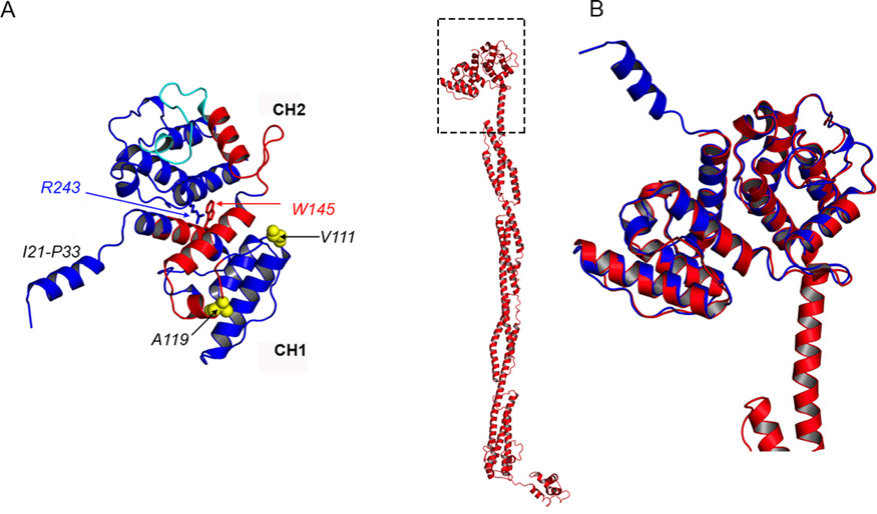
Crystal structure obtained for the WT ABD of ACTN2 and a comparison between this structure and that obtained for the almost full length protein reported recently [13] (**A**) Our crystal structure of the WT ABD of ACTN2 with the CH1 domain shown at the bottom, and the CH2 domain at the top. Red indicates the identified actin binding regions and cyan the PIP_2_ binding region. The structure contains an additional resolved N-terminal α-helix (I21-P33). The stacking of Arg^243^ and Trp^145^ between CH1 and CH2 is indicated. (**B**) Superposition of the WT1 ABD of ACTN2 (blue) on to the ABD of the full length ACTN2 (red) (PDB code 4D1E [10]).

In the closed conformation that we observed, there is extensive contact between the CH1 and CH2 domains, and the residues Arg^243^ and Trp^145^ stack together (Figure 3A), as found for other ACTN ABDs [26,33,34]. The stacking of Arg^243^ and Trp^145^ is similar to the stacking between the equivalent lysine residue at position 255 in the CH2 domain of ACTN4 with tryptophan (135) in the closed conformation. Interestingly, the crystal structure of the K255E mutant of the ACTN4 ABD also showed a closed structure, even though this mutation was predicted to promote an open conformation [34]. Although the ACTN2 ABD may adopt a more open conformation, in which the two CH domains become more separated when it binds to actin [35], currently there is no clear demonstration that this does happen.

Intriguingly, one of our two structures for the WT construct contains an additional N-terminal α-helix, upstream of the first CH1 domain not seen previously (Figure 3A). From our structures of the other WT and the two mutants that do not have this N-terminus helix, this may suggest that the N-terminus is highly flexible. The appearance of this helix for one of our structures may suggest that in this case, the crystal packing stabilizes the helix by adjacent molecules. This helix is not thought to be involved in binding to actin but it could be involved in binding to one of the many other Z-disc proteins known to bind to α-actinin (reviewed in [31]). Muscle LIM protein is known to bind close to this region, and the Q9R mutation in ACTN2 has been shown to interfere with its ability to bind muscle LIM protein [36]. Aligning this structure of the WT ACTN2 with that for the almost full length ACTN2 recently published [13] shows how this N-terminal helix points away from the CH domains, but that otherwise our structure super-imposes well (Figure 3B).

**Figure 4:**
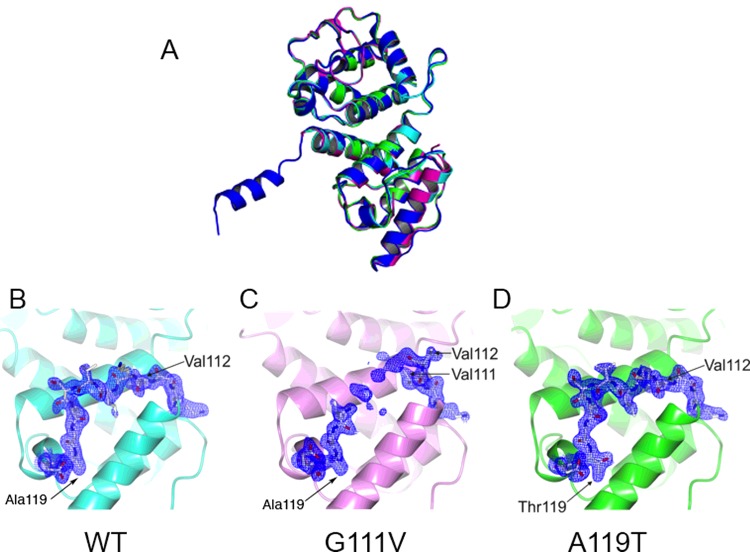
Effects of mutations in the actin binding domain on structure (**A**) Overlay of the WT1 (blue), WT2 (cyan), G111V (magenta) and A119T (green) mutant ABD of ACTN2 structures. (**B**–**D**) 2F_o_-F_c_ electron density contoured at 0.9*σ* for the loop regions 110–120 for WT and the two mutant constructs.

Super-imposing the structures for the two mutants on to the WT structure for the ABD of ACTN2 derived from a crystal structure for the almost full length protein (residues 34–892 were assigned in the deposited model [13]) shows only minor differences in the region of overlapping structure (Figures 4A–4D). However, although continuous densities are observed for the loop region at residues 110–120 in both the WT (Figure 4B) and the A119T mutant structure (Figure 4D), this region is disordered for G111V mutant between residues 112 and 117 (Figure 4C). For both the WT and A119T mutant structure, Val^112^ is orientated towards the interior of the molecule packing the side chain into a hydrophobic region. For the G111V mutant, the mutant residue Val^111^ now occupies this same hydrophobic region and thus reorients Val^112^ and the subsequent residues away from the molecule resulting in a more flexible conformer for this loop region. This larger alteration to the structure for G111V may help to explain the decreased thermal stability for this mutation observed by circular dichroism (Figure 2B).

The CH domains of ACTN are thought to have three regions important in binding to actin: residues 41–50, 116–140 and 145–165 (reviewed in [31]). G111V is just outside of one of these regions, and although this mutation appears to introduce some instability into the subsequent loop containing this binding region, it may not have a large effect on actin binding affinity. A119T is within the binding region itself, and its alterations to the structure may be expected to have a small effect on binding. These observations may explain why, despite the small changes in structure, we observed a larger effect of the G111V mutation on thermal stability and tertiary structure, whereas A119T had a larger effect on actin binding.

### Mutations in the CH domains in ACTN2 reduce incorporation into Z-discs in cardiomyocytes

**Figure 5:**
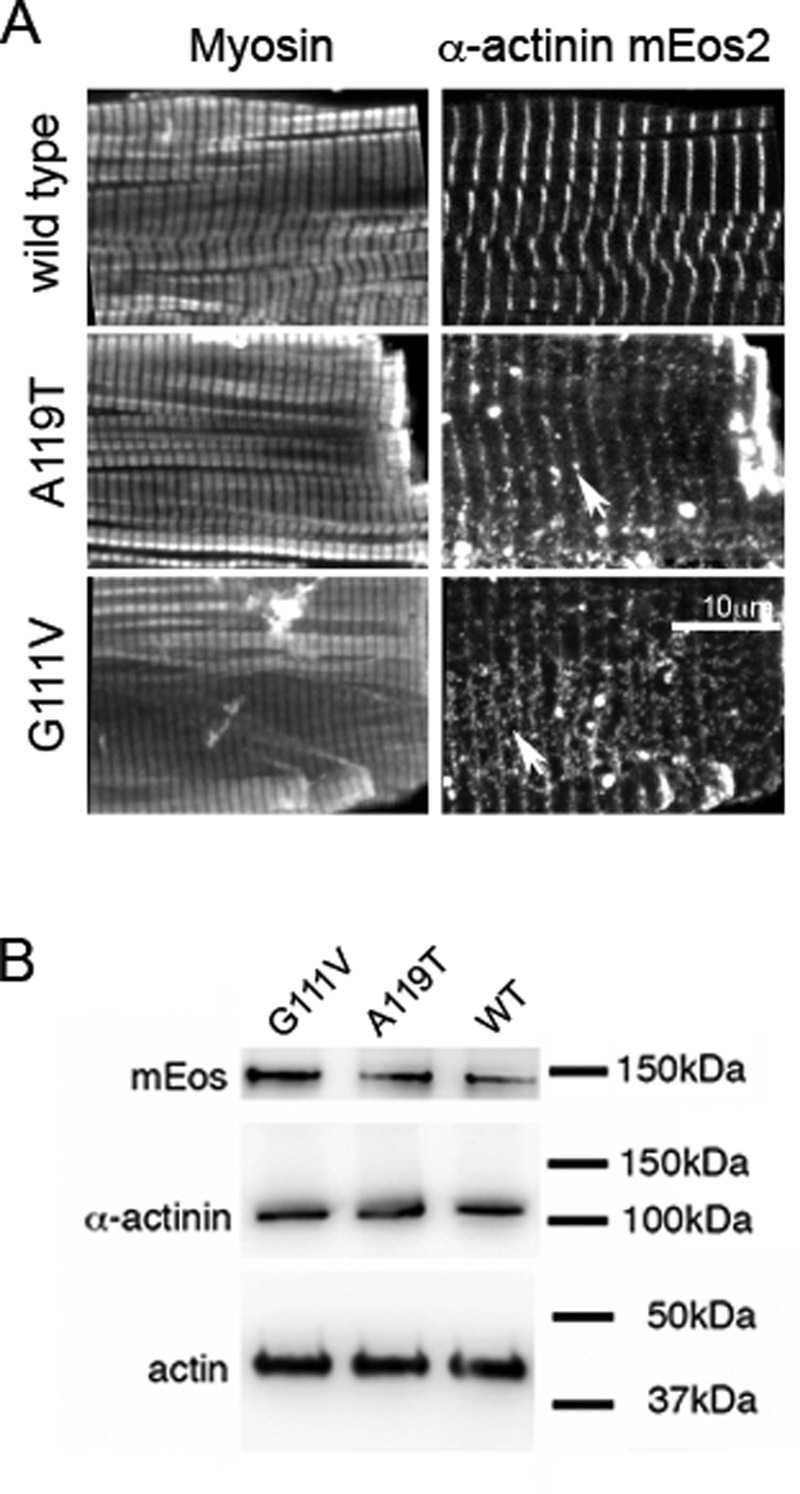
Expression of wild type and mutant isoforms of mEos2-tagged ACTN2 in adult cardiomyocytes (**A**) Cells were co-stained for myosin heavy chain. Arrows for the mutant isoforms in the ACTN2 mEos2 images shows the presence of aggregated protein in addition to Z-disc localization. These images are representative images obtained for three separate experiments. Scale bar: 10 μm. (**B**) Western blots for adult cardiomyocytes expressing mEos2-tagged ACTN2. Anti-mEos2 bands show comparative expression levels for all three constructs. Anti-ACTN2 did not detect an additional band for the mEos2-tagged protein, due to low levels of expression. Anti-actin shows similar loading for each sample.

WT, A119T and G111V mEos-2 tagged human ACTN2 all localized to Z-discs in adult cardiomyocytes (Figure 5A). However, the integration of mutant ACTN2 appeared somewhat weaker than that for WT ACTN2, and there was a greater tendency for mutant isoforms to form aggregates outside the Z-discs. Expression levels of the mutant isoforms were similar to those of WT ACTN2 (Figure 5B). Therefore, it is unlikely that the change in localization observed for the mutant isoforms is a result of large differences in expression levels.

**Figure 6:**
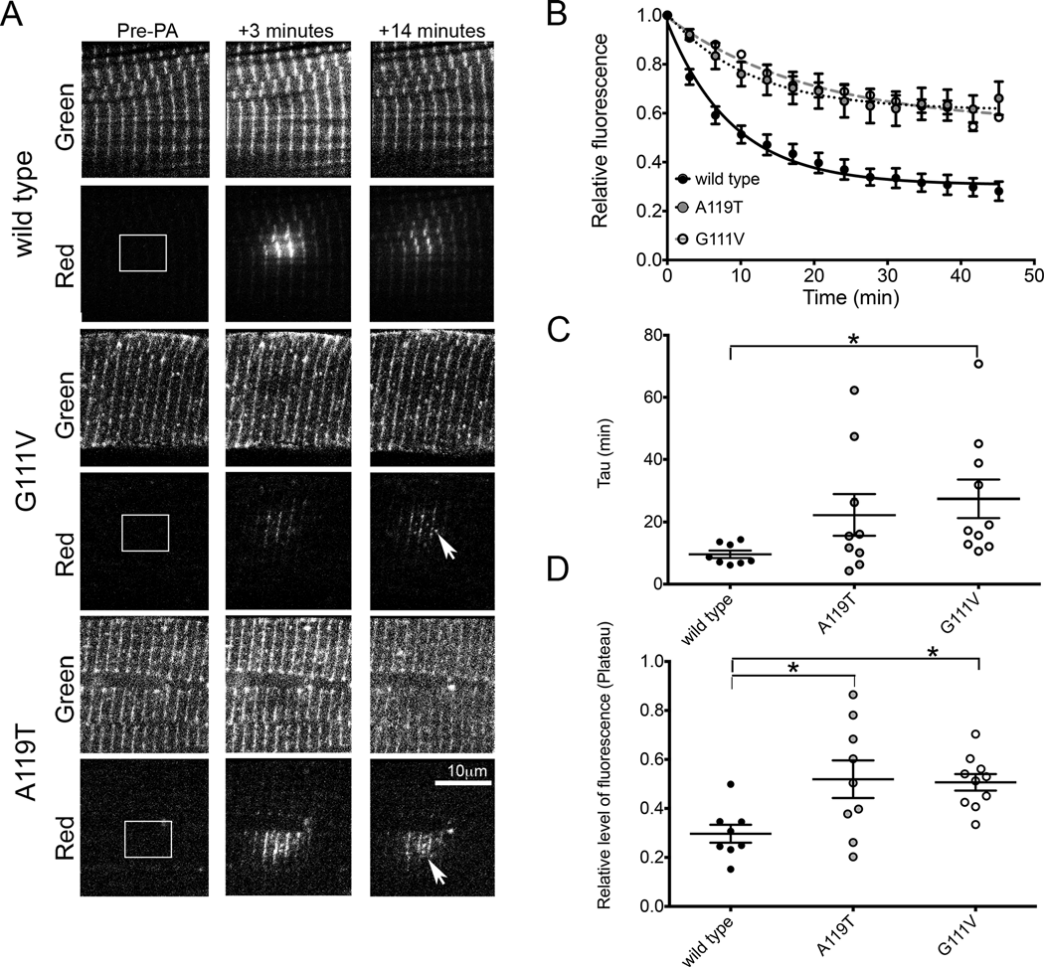
Dynamic behaviour of wild type and mutant mEos2-tagged ACTN2 in live adult cardiomyocytes (**A**) A small region of the cell (indicated by the white box in the pre-photoactivation images [Pre-PA)] is illuminated with 405 nm light, which results in the photoconversion of ‘green’ mEos2-ACTN2 in this region to red, as shown in the adjacent image at +3 min after photoconversion. The red-fluorescent protein can then diffuse away from the initial site of activation (as shown in subsequent frame at +14 min). Examples for WT and the two mutant constructs are shown. (**B**) The change in fluorescence in the central region of photoactivation is plotted against time to show the decay of fluorescence. (Values shown are the means±S.E.M. where for WT, *n*=8; AT mutant, *n*=9; and GV mutant, *n*=10.) (**C**) Box and Whisker plot for measurements of *τ*, for the rate of change of fluorescence in (**B**) for WT and the mutant constructs. Mean±S.E.M. values are overlaid on the individual measurements. (**D**) Box and Whisker plot for measurements of the plateau levels of fluorescence for WT and mutant constructs, calculated from the fits to the curve in (**B**). Mean±S.E.M. values are overlaid on the individual measurements (*n*=8 cells for WT, 9 for AT and 10 for G111V, and measurements were obtained from cells from two separate experiments for each construct). Levels of statistical significance: *P<0.05.

In addition, both mutant isoforms affected the dynamic behaviour of ACTN2. After photoactivation of a small region of mEos2-tagged ACTN (Figure 6A), we found that the rate of fluorescence decay from the central region of the ‘photoswitched’ area was reduced for both G111V and A119T mutants compared with WT ACTN2 (Figures 6B–6D), and this change was significant for G111V. This suggests that mutant ACTN that does incorporate into the Z-disc is less able to leave (Figure 6D). Taken together, these data suggest that the exchange of mutant protein within the the Z-disc is aberrant for both mutants compared with WT ACTN2.

These data show that both mutations appear to reduce the ability of the protein to incorporate normally into Z-discs. The small reduction in actin binding affinity we observed *in vitro* for the isolated CH domains for the two mutants may help to account for the reduction in incorporation of the full length mEos2-tagged ACTN into Z-discs in cells, although the changes we observed *in vitro* were small and non-significant. If the mutant full length proteins are less able to incorporate into the Z-discs, then the unincorporated protein may be more likely to aggregate as we observed. As ACTN2 is important for structural integrity and mechanosensing of the Z-disc, reduced incorporation could lead to reduced force transmission in cardiac muscle, thus providing a potential mechanism for development of disease.

In the full length protein, the C-terminal EF3/4 domain of ACTN2 would be expected to be more important in localizing ACTN2 to the Z-disc, as it binds tightly to the Z-repeat 7 (ZR7) of titin (Figure 2B). However, the slightly weaker actin binding affinity that we measured for both mutants does appear to affect the dynamic incorporation of ACTN2 into Z-discs. The binding of ACTN2 to ZR7 depends on the binding of PIP_2_ to the ABD, which results in the release of the EF3/4 domain from the neck (Figure 2), allowing the EF3/4 domain to bind to ZR7. PIP_2_ is thought to bind to a triplet of residues Arg^163^, Arg^169^ and Arg^192^ [26]. The binding affinity of the neck domains for ZR7 in the presence of PIP_2_ is ∼10-fold higher (*K*_d_ of 380 nM [13]) than in its absence, and over 10-fold higher than the binding affinity of the CH domains for actin. PIP_2_ binding mutations in ACTN do not affect F-actin binding [13], and it is unlikely that the ACTN mutations we tested here affect PIP_2_ binding. We speculate that perhaps there is some kind of mechanical strain between the actin and titin binding sites in ACTN2, when it is localized to the Z-disc, which promotes the exchange of ACTN2 between Z-discs. A reduced actin binding capability may reduce this strain, thereby reducing the rate and extent of exchange of ACTN molecules in the Z-disc.

As discussed in the Introduction, the two mutations we have investigated here result in a relatively mild phenotype, are likely to be benign, and in common with many HCM mutations in sarcomeric proteins, may take many years to show themselves as an obvious phenotype [4]. Our data suggest that both G111V and A119T mutations affect the ability of ACTN2 to function normally. Although these mutations have only small effects on structure and behaviour, this is consistent with their mild phenotype, and thus they are likely to be genuine disease mutations.

## Abbreviations

ABD: actin binding domain
ACTN2: α-actinin-2
CH: calponin homology
GST: glutathione transferase
HCM: hypertrophic cardiomyopathy
MR: molecular replacement
MTS: multi-time series
PIP_2_: PtdIns(4,5)*P*_2_
ROI: region of interest
WT: wild type
ZR7: Z-repeat 7
